# Inflation Reduction Act Provisions and Medicare Part D Out-of-Pocket Costs for Specialty Drugs

**DOI:** 10.1001/jamahealthforum.2025.1387

**Published:** 2025-05-16

**Authors:** Jalpa A. Doshi, Pengxiang Li, Matthew J. Klebanoff, John K. Lin

**Affiliations:** 1 Division of General Internal Medicine, Perelman School of Medicine, University of Pennsylvania, Philadelphia; 2 Leonard Davis Institute of Health Economics, University of Pennsylvania, Philadelphia; 3 Division of Cancer Medicine, The University of Texas MD Anderson Cancer Center, Houston

## Abstract

This cross-sectional study examines the relative association of US Inflation Reduction Act (IRA) provisions with out-of-pocket costs for specialty drugs for Medicare Part D beneficiaries.

## Introduction

Specialty drugs represent major treatment advances, albeit accompanied by a “too much, too soon” out-of-pocket (OOP) cost burden for Medicare Part D beneficiaries.^1^ The Inflation Reduction Act (IRA) included 3 provisions that could reduce Part D OOP costs for beneficiaries using specialty drugs^2^: (1) an annual Part D OOP maximum ($2000 beginning 2025 and indexed by program growth thereafter), (2) a voluntary Medicare Prescription Payment Plan (MPPP) beginning 2025 that helps “smooth” (ie, spread out) Part D OOP costs throughout the year via monthly payments,^3^ and (3) negotiated drugs prices starting 2026.^4^ Although these provisions hold promise for lowering specialty drug OOP costs, the relative contribution of each provision is unknown.

## Methods

In this study, OOP costs were estimated for the 3 specialty drugs (etanercept, ustekinumab, ibrutinib) selected for 2026 drug price negotiation (eTable in Supplement 1).^4^ Annual OOP costs were calculated using 2023 list prices and 2026 negotiated prices.^4^ We estimated annual OOP costs using standard Part D benefit parameters for beneficiaries without low-income subsidies in 2023 (ie, before the redesigned Part D benefit) and 2026 (ie, after all 3 IRA provisions took effect) (eMethods in Supplement 1). We also examined how monthly OOP costs in 2026 varied based on the decision to enroll in the MPPP or not.^5^ We estimated OOP costs assuming use of the specialty drug alone because these drugs account for more than 90% of total annual OOP costs among beneficiaries who use them.^1^ This study followed the STROBE reporting guidelines and, per the Common Rule, was exempt from ethics review because it used publicly available data.

## Results

In 2023, with pre-IRA list prices, Medicare beneficiaries had annual OOP costs of $6807 (etanercept), $10 845 (ustekinumab), and $11 504 (ibrutinib) (Table).^4^ If the IRA’s lower prices had been in effect in 2023 (without the annual OOP maximum), beneficiaries would have had annual OOP costs of $3957 (etanercept), $5361 (ustekinumab), and $8135 (ibrutinib).

**Table.** Estimated Annual OOP Costs in 2023 and 2026 for Medicare Part D Beneficiaries Using High-Cost Specialty Drugs Selected for Medicare Drug Price Negotiation

Characteristic	Etanercept	Ustekinumab	Ibrutinib
Indications	Rheumatoid arthritis, psoriasis, psoriatic arthritis, ankylosing spondylitis	Psoriasis, psoriatic arthritis, Crohn disease, ulcerative colitis	Chronic lymphocytic leukemia, Waldenström macroglobulinemia, chronic graft-vs-host disease
List price for 30-d supply without Medicare drug price negotiation, $^a^	7106	13 836	14 934
Annual OOP costs under 2023 Medicare Part D benefit, $^b^	6807	10 845	11 504
Annual OOP costs under 2026 Medicare Part D benefit, $^c^	2100	2100	2100
Negotiated price for 30-d supply after Medicare drug price negotiation, $^a^	2355	4695	9319
Annual OOP costs under 2023 Medicare Part D benefit, $^b^	3957	5361	8135
Annual OOP costs under 2026 Medicare Part D benefit, $^c^	2100	2100	2100

^a^ The prices were obtained from the Center for Medicare & Medicaid Services announcement on the Medicare Drug Price Negotiation Program’s negotiated prices for initial price applicability year 2026^4^; list price represents the price for a 30-day supply in 2023 as reported in the announcement.

^b^ The final standard Part D benefit parameters for 2023 include a $505 deductible followed by a 25% coinsurance until reaching the catastrophic threshold (up to $7400 in out-of-pocket [OOP] costs), at which point beneficiaries pay 5% coinsurance until end of the calendar year.

^c^ The final Medicare Part D benefit parameters for 2026 include a $615 deductible followed by a 25% coinsurance until reaching the $2100 annual OOP maximum.

In 2026, the IRA will lower annual OOP costs for each specialty drug to $2100. The annual OOP cost would be the same even if higher pre-IRA 2023 list prices were still in effect. In 2026, beneficiaries who do not enroll in the MPPP will face higher OOP costs for their first prescription fill in January ($1050 [etanercept], $1635 [ustekinumab], and $2100 [ibrutinib]) compared with those enrolled in the MPPP ($175) and will reach their $2100 OOP limit with 3, 2, and 1 thirty-day fills, respectively (Figure).

**Figure.**
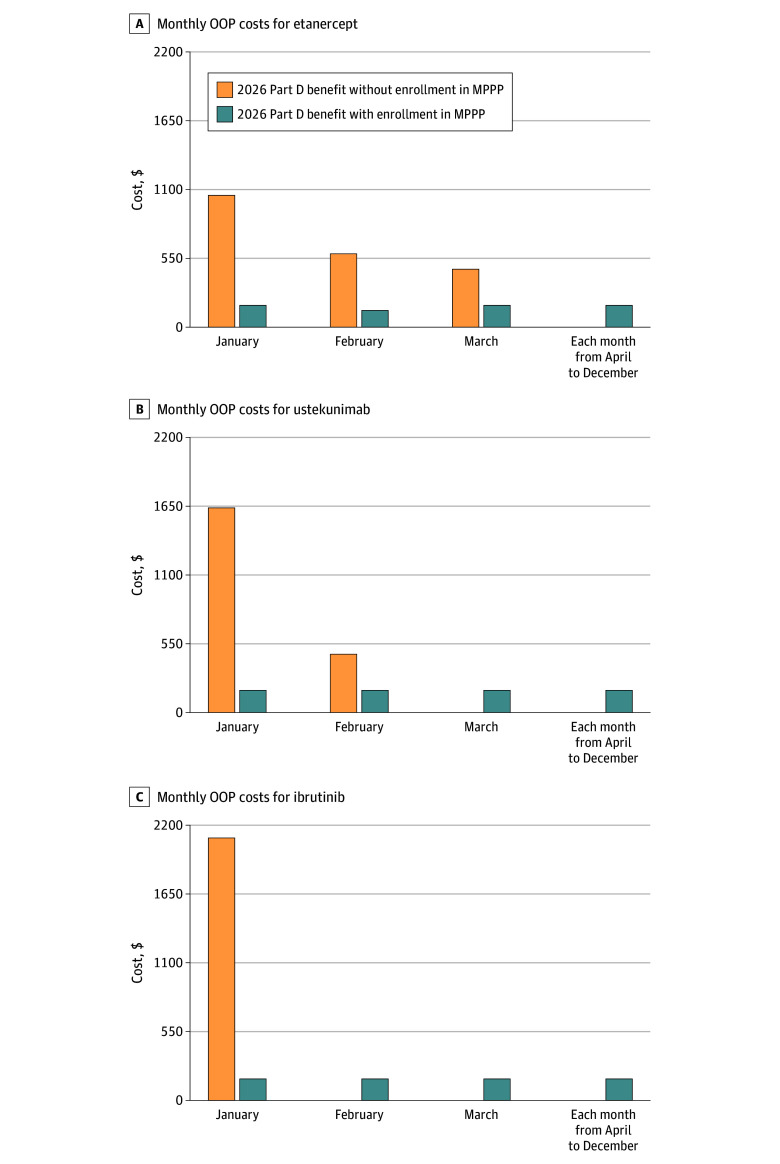
Estimated Monthly Out-of-Pocket (OOP) Costs in 2026 for Medicare Part D Beneficiaries Using High-Cost Specialty Drugs Selected for Medicare Drug Price Negotiation With and Without Enrollment in the Medicare Prescription Payment Plan (MPPP) Medicare Part D beneficiaries can enroll in the MPPP at any point in the calendar year. Whenever beneficiaries enroll in the MPPP, the first month’s maximum monthly cap is calculated by subtracting a beneficiary’s total OOP spending to date from the annual OOP maximum ($2100 in 2026) and dividing by the months remaining in the plan year. The maximum monthly cap for subsequent months is calculated by dividing the remaining OOP cost from previous months (plus any additional OOP incurred by the beneficiary) by the months remaining in the plan year. In this figure, monthly OOP costs under the 2026 Part D benefit with MPPP enrollment are estimated for a beneficiary enrolling in this voluntary program in January. Although patients can enroll in the MPPP at any point in the calendar year, beneficiaries using specialty drugs will reap the most benefit if they enroll in the MPPP earlier in the year, as they will have more months to spread out their OOP costs. For a patient who initiates a specialty drug in November and then enrolls in the MPPP, their monthly OOP cost could be as high as $1050 (ie, $2100 annual OOP maximum divided by two months remaining in the year) assuming they had no Part D expenses earlier in the year. However, starting January of the following year, their OOP costs will be only $175 per month ($2100 annual OOP max/12 calendar months), as shown.

## Discussion

For Part D beneficiaries using specialty drugs, price negotiation will lead to a relatively smaller reduction in annual Part D OOP costs compared with the IRA’s annual OOP maximum. Beneficiaries using specialty drugs will experience a large reduction in OOP costs only with implementation of the IRA’s annual OOP maximum. Although price negotiation has a relatively small contribution to lowering OOP costs, it will enable savings to the Medicare program necessary to offset the costs of the new OOP maximum.

Our findings highlight large differences in monthly Part D OOP costs based on beneficiaries’ enrollment in the MPPP. Beneficiaries who do not enroll will face substantial OOP costs at the start of the year, even with the annual OOP maximum in effect. For ibrutinib, the entire annual OOP maximum ($2100) would come due for the first prescription in January; such high costs have been associated with high rates of prescription abandonment.^6^ Based on how MPPP calculations work, enrollment earlier in the year could reduce this frontloaded OOP burden by distributing costs evenly over the remaining year.^5^

Study limitations include necessary assumptions for our calculations, such as only accounting for OOP costs for specialty drugs; thus, our calculations may have underestimated OOP costs in 2023, and beneficiaries may reach the $2100 limit more quickly in 2026 than our estimates suggest. Nonetheless, the government, clinical societies, and media organizations should promote the MPPP for patients using specialty drugs, to ensure maximal benefit from the IRA’s Part D OOP provisions.

## Notes

Supplement 1. **eTable.** List Price and Negotiated Price for the 10 Medicare Part D Drugs Selected for the First Cycle of Medicare Drug Price Negotiations for Initial Price Applicability Year 2026
eMethods.

Supplement 2.
Data Sharing Statement
