# Supplementary material for: Inflation Reduction Act Provisions and Medicare Part D Out-of-Pocket Costs for Specialty Drugs
Source: JAMA Health Forum. 2025 May 16;6(5):e251387. doi: 10.1001/jamahealthforum.2025.1387 (PMC12084838; doi:10.1001/jamahealthforum.2025.1387)
Supplement: Supplement 1. — eTable. List Price and Negotiated Price for the 10 Medicare Part D Drugs Selected for the First Cycle of Medicare Drug Price Negotiations for Initial Price Applicability Year 2026 eMethods. [file jamahealthforum-e251387-s001.pdf]

Supplementary Online Content

Doshi JA, Li P, Klebanoff MJ, Lin JK. Inflation Reduction Act provisions and Medicare Part D out-of-pocket costs for specialty drugs. *JAMA Health Forum.* 2025;6(5):e251387. doi:10.1001/jamahealthforum.2025.1387

eTable. List Price and Negotiated Price for the 10 Medicare Part D Drugs Selected for the First Cycle of Medicare Drug Price Negotiations for Initial Price Applicability Year 2026

eMethods.

This supplementary material has been provided by the authors to give readers additional information about their work.

© 2025 Doshi JA et al. *JAMA Health Forum.*

eTable. List Price and Negotiated Price for the 10 Medicare Part D Drugs Selected for the First Cycle of Medicare Drug Price Negotiations for Initial Price Applicability Year 2026

**Drug Name (Brand Name)**	**Commonly Treated Conditions**	**List price for 30-day supply, CY 2023**	**Agreed to Negotiated Price for 30- day Supply for CY 2026**	**Specialty drug**
Ibrutinib (Imbruvica®)	Mantle cell lymphoma, chronic lymphocytic leukemia, Waldenström's macroglobulinemia	$14,934	$9,319	Yes
Ustekinumab (Stelara®)	Crohn's disease, ulcerative colitis, plaque psoriasis, psoriatic arthritis	$13,836	$4,695	Yes
Etanercept (Enbrel®)	Rheumatoid arthritis, psoriatic arthritis, ankylosing spondylitis, plaque psoriasis	$7,106	$2,355	Yes
Sacubitril/valsartan (Entresto®)	Heart failure	$628	$295	No
Empagliflozin (Jardiance®)	Type 2 diabetes, heart failure	$573	$197	No
Dapagliflozin (Farxiga®)	Type 2 diabetes, heart failure, chronic kidney disease	$556	$179	No
Sitagliptin (Januvia®)	Type 2 diabetes	$527	$113	No
Apixaban (Eliquis®)	Atrial fibrillation, deep vein thrombosis, pulmonary embolism	$521	$231	No
Rivaroxaban (Xarelto®)	Atrial fibrillation, deep vein thrombosis, pulmonary embolism	$517	$197	No
Insulin aspart (Fiasp®)	Type 1 and Type 2 diabetes	$495	$119	No

Source: CMS Announcement of Negotiated Prices: www.cms.gov/files/document/fact-sheet-negotiated-prices-initial-price-applicability-year-2026.pdf

© 2025 Doshi JA et al. *JAMA Health Forum.*

eMethods.

1. *Data Source*

The data sources used for calculating the OOP costs in this study are the CMS announcement of the negotiated Medicare drug prices and publicly available standard Medicare Part D benefit parameters. Both of these sources are publicly available at the links below:

- CMS Announcement of Negotiated Prices: www.cms.gov/files/document/fact-sheet-negotiated-prices-initial-price-applicability-year-2026.pdf
- CMS Yearly Announcements of Medicare Part D Parameters: https://www.cms.gov/medicare/payment/medicare-advantage-rates-statistics/announcements-and-documents

2. *Drug Selection*

Our focus was specifically on how the different provisions of the IRA will impact the OOP cost burden of patients using the three specialty drugs (etanercept, ustekinumab, ibrutinib) that have list prices multiple-folds higher than the other seven brand-name drugs selected for price negotiation (see eTable 1). Virtually all Medicare Part D plans place the three specialty drugs on a specialty tier and subject them to coinsurance (25% to 33%) calculated based on the drugs' list prices. Such specialty drugs have been shown to specifically present a "too much too soon" out-of-pocket cost burden for Medicare beneficiaries under the Part D benefit in the pre-IRA period (Doshi et al., 2017). In other words, Part D beneficiaries spent thousands of dollars each year for their specialty drug alone ("too much" problem) and these costs were typically "frontloaded" at the start of each calendar year ("too soon" problem). The purpose of this research letter was to examine the relative impact of the key provisions of the IRA on these OOP cost issues faced by specialty drug users.

3. *Selecting Medicare Part D Benefit Parameters*

Per the IRA legislation, any drug that undergoes negotiation must be covered on formularies of every Medicare Part D plan (KFF 2023). Thus, there will be little variation in the actual formulary coverage of the three specialty drugs examined in our study. The only source of variation would arise from the individual Part D plan benefit parameters. For our primary analysis, we applied the standard Medicare Part D benefit including a deductible and 25% coinsurance. However, Part D plans are permitted to offer alternative Part D benefit designs that are either actuarially equivalent or enhanced (e.g., no deductible) relative to the standard Part D benefit. Regardless of plan type, the vast majority of Part D plans require a deductible, place specialty drugs on specialty tiers, and subject them to coinsurance ranging from 25% to 33% (KFF 2024). In our primary analysis we assumed the standard deductible (e.g., $505 in 2023) and coinsurance (25%). However, we also conducted sensitivity analyses to identify the range of annual OOP estimates based on the most generous plan offering ($0 deductible + 25% coinsurance) and least generous plan offering (standard deductible + 33% coinsurance). The annual OOP cost estimates under the pre-IRA 2023 drug benefit were only up to 4% lower for the most generous plan and up to 12% higher for the least generous plan relative to our primary estimates across the three specialty drugs. The annual OOP cost estimates for the post-IRA 2026 estimates remained unchanged. Hence, given the small differences, we only present results for our primary analysis using the standard Part D benefit parameters.

The final standard Part D benefit parameters for 2023 included a $505 deductible followed by a 25% coinsurance until reaching the catastrophic threshold (up to $7,400 in TrOOP costs), at which point beneficiaries pay 5% coinsurance until end of the calendar year. The final Medicare Part D benefit parameters for 2026 included a $615 deductible followed by a 25% coinsurance until reaching the $2,100 OOP maximum.

4. *Calculating Monthly Costs with Medicare Prescription Payment Plan (MPPP)*

The plan year for all Medicare Part D beneficiaries starts on January 1 and ends on December 31. The plan benefit resets on January 1 of the next year. Per the IRA, patients can enroll in the MPPP at any point during the calendar year. For beneficiaries who enroll in the MPPP, the first month's maximum monthly cap is calculated by subtracting a beneficiary's total OOP spending to date from the annual OOP maximum ($2,100 in 2026) and dividing by the months remaining in the plan year. The maximum monthly

© 2025 Doshi JA et al. *JAMA Health Forum.*

cap for subsequent months is calculated by dividing the remaining OOP from previous months (plus any additional OOP incurred by the beneficiary) by the months remaining in the plan year.

Based on the above formulae, it is clear that beneficiaries using specialty drugs will reap the most benefit if they enroll in the MPPP earlier in the year as they will have more months to “smooth” out their OOP costs. The monthly OOP costs under the 2026 Part D benefit with MPPP enrollment in our study were estimated assuming a beneficiary enrolling in this voluntary program in January. For a patient who initiates a specialty drug in November and then enrolls in the MPPP, their monthly OOP cost could be as high as $1,050 (i.e., $2,100 annual OOP maximum divided by two months remaining in the year) assuming they had no Part D expenses earlier in the year. However, starting January of the following year, their OOP costs will be similar to our calculated estimates for a beneficiary enrolling in this voluntary program in January (i.e. annual OOP max / 12 calendar months).

5. Assumptions and Rationale for OOP calculations

Our analysis was meant to be illustrative and assumed that Medicare Part D patients who do not receive a low-income subsidy (non-LIS patients) initiated the specialty drug in January (i.e., at the start of the plan year) and filled prescriptions continuously (the 3 specialty drugs are to be taken long-term until disease progression or intolerance) over the entire calendar year. Our analysis focused on OOP costs for the specialty drug alone given that prior work has established that specialty drugs account for greatest source of Part D costs for patients receiving specialty drug treatment. We also assumed that beneficiaries faced the standard Part D benefit (see rationale in #3 above) and were not receiving additional help to assist paying their OOP costs.

© 2025 Doshi JA et al. *JAMA Health Forum.*
