# Supplementary material for: Inflation Reduction Act Provisions and Medicare Part D Out-of-Pocket Costs for Specialty Drugs
Source: JAMA Health Forum. 2025 May 16;6(5):e251387. doi: 10.1001/jamahealthforum.2025.1387 (PMC12084838; doi:10.1001/jamahealthforum.2025.1387)
Supplement: Supplement 2. — Data Sharing Statement [file jamahealthforum-e251387-s002.pdf]

Data Sharing Statement

Doshi. Inflation Reduction Act Provisions and Medicare Part D Out-of-Pocket Costs for Specialty Drugs. *JAMA Health Forum*. Published May 16, 2025. doi:10.1001/jamahealthforum.2025.1387

Data
Data available: Yes
Data types: Data (not involving human participants)
How to access data: Data is publicly available via CMS announcements. Links are provided in our letter to both the 2023 list price and 2026 negotiated price as well as the Part D benefit changes enacted by the IRA. Negotiated Prices: https://www.cms.gov/newsroom/fact-sheets/medicare-drug-price-negotiation-program-negotiated-prices-initial-price-applicability-year-202 Medicare Benefit Changes: https://www.cms.gov/inflation-reduction-act-and-medicare/part-d-improvements
When available: With publication

Supporting Documents
Document types: None

Additional Information
Who can access the data: Anyone with the link can access the data
Types of analyses: For any purpose
Mechanisms of data availability: Anyone with the link can access the data
Any additional restrictions: N/A
